# Physicochemical Characterization of *Camellia oleifera* Husks from Different Regions and Microwave-Assisted RSM Optimization of Tea Saponin Extraction

**DOI:** 10.3390/foods14193380

**Published:** 2025-09-29

**Authors:** Weixian Wu, Yuhuan Liu, Jian Huang, Xiaoyan Liu, Guangda Zhang, Zhiqiang Gu, Shuangquan Huang, Yunpu Wang, Qi Zhang

**Affiliations:** 1 Engineering Research Center for Biomass Conversion, Ministry of Education, Nanchang University, Nanchang 330047, China; 417900230078@email.ncu.edu.cn (W.W.); 417900240165@email.ncu.edu.cn (X.L.); zhanggungda@email.ncu.edu.cn (G.Z.); guzhiqiang0816@email.ncu.edu.cn (Z.G.); 2 State Key Laboratory of Food Science and Resources, Nanchang University, Nanchang 330013, China; liuyuhuan@ncu.edu.cn (Y.L.); wangyunpu@ncu.edu.cn (Y.W.); 3 School of Business Administration, Jiangxi University of Finance and Economics, Nanchang 330047, China; 4 Jiangxi Provincial Enterprise Technology Center, Yichun 330804, China; 352313320026@email.ncu.edu.cn

**Keywords:** microstructure, *Camellia oleifera* husks, chemical composition, liquid-to-solid ratio, ethanol concentration, extraction time, microwave power

## Abstract

This study investigated the physicochemical properties of *Camellia oleifera* husks collected from three regions of Jiangxi Province (Ganzhou—GZ, Yichun—YC, and Jiujiang—JJ) and extracted tea saponins via microwave-assisted solvent extraction (MASE), aiming to provide a theoretical basis for the high-value utilization of this agricultural by-product. The husks from YC were rich in bioactive compounds such as tea saponins (16.29 ± 0.02%), with lower cellulose (21.05 ± 1.05%) and lignin (12.48 ± 1.14%) contents and higher hemicellulose (27.40 ± 0.80%) content. The husks from JJ exhibited abundant porosity and a larger specific surface area (40–60 mesh, 4.15 ± 0.04 m^2^/g). Single-factor extraction experiments indicated that the microstructure and chemical composition of *Camellia oleifera* husks significantly affected the extraction efficiency of saponins, tannins, and flavonoids. The optimal extraction conditions for tea saponins were established using Box–Behnken response surface methodology, with the liquid-to-solid ratio identified as the most critical factor. Optimal conditions for GZ husks were a liquid-to-solid ratio of 46.75 mL/g, ethanol concentration of 35.5%, extraction time of 6 min, and microwave power of 350 W, with the extraction yield of 7.49 ± 0.01%. Optimal conditions for YC husks were a liquid-to-solid ratio of 50.55 mL/g, ethanol concentration of 40.13%, extraction time of 6 min, and microwave power of 350 W, with the extraction yield of 16.29 ± 0.02%. Optimal conditions for JJ husks were a liquid-to-solid ratio of 47.44 mL/g, ethanol concentration of 37.28%, extraction time of 6 min, and microwave power of 350 W, with the extraction yield of 9.39 ± 0.02%. The study provides important scientific evidence for understanding the structure–function relationship of *Camellia oleifera* husks and offers practical guidance for developing sustainable industrial processes to convert agricultural by-products into high-value bioactive compounds, thereby promoting resource recycling and economic benefits in the *Camellia oleifera* industry.

## 1. Introduction

*Camellia oleifera*, belonging to the family *Theaceae* and genus *Camellia*, is a vital woody oil crop in China and serves as the source of tea seed oil [1]. Thriving in acidic soils, it is a subtropical evergreen shrub widely cultivated in countries including China, Brazil, India, and South Korea [2]. In China, *C. oleifera* is extensively distributed across over 1100 counties in 15 provinces (regions) throughout Central, South, East, and Southwest China [3]. The country possesses abundant *C. oleifera* resources, with a cultivation area reaching 4.2667 million hectares—accounting for over 80% of the nation’s total woody edible oil crop area [4,5]. Amid agricultural restructuring and the implementation of the national Grain for Green Program, *C. oleifera* stands out due to its dual advantages as an economic and ecological forest [6]. Its marked industrial advantages and economic competitiveness make it one of the fastest-growing and most promising woody oil crops in China [7]. While the *C. oleifera* industry drives economic growth in southern China, it also generates millions of tons of *C. oleifera* husk by-products annually [8]. To manage this volume, the husks are often disposed of as biological waste through landfilling or incineration, resulting in significant resource wastage and environmental pollution.

The calorific value of *C. oleifera* husks is relatively high (about 16–18 MJ/kg), close to that of lignite, making it an excellent biomass fuel. *C. oleifera* husks primarily consist of hemicellulose (25–35%), lignin (20–30%), and cellulose (15–25%). They are also rich in various bioactive compounds [9], such as tea saponins, tannins, polysaccharides, and flavonoids. Tea saponins, a group of triterpenoid glycosides mainly derived from *Camellia oleifera* by-products, have attracted considerable attention owing to their diverse biological activities and industrial applications [10]. They exhibit multiple surface-active properties, including emulsification, dispersion, wetting, and foaming, and possess remarkable pharmacological effects such as antimicrobial, antiviral, antioxidant, anti-inflammatory, hypolipidemic, and antitumor activities [11]. Because they are natural, phosphorus-free, and non-ionic surfactants, tea saponins are widely used in the chemical, food, pharmaceutical, and agricultural industries, highlighting the necessity of developing efficient extraction strategies with high yield and purity [12].

To date, various methods have been employed for tea saponin extraction, such as hot water extraction, water–ethanol precipitation, Soxhlet extraction, ultrasonic-assisted extraction, macroporous resin adsorption, supercritical CO_2_ extraction, and foam separation [13], with each method presenting certain advantages while facing notable limitations. For instance, hot water extraction is cost-effective and environmentally friendly [14], yet it often co-extracts sugars, proteins, and polysaccharides, resulting in low purity and difficulties in downstream purification. Water–ethanol precipitation improves purity but requires significant solvent consumption and investment [15], with ethanol volatility being a safety concern. Ultrasonic-assisted extraction effectively reduces extraction time and temperature [16], minimizing thermal degradation, but generally requires a combination with other techniques for higher efficiency. Macroporous resin and supercritical CO_2_ extraction provide selective enrichment with good product quality; however, these methods are highly sensitive to process parameters and demand costly equipment [17]. Foam separation demonstrates a high yield, yet its performance is strongly influenced by airflow and temperature, limiting reproducibility and scalability [18].

Among emerging approaches, microwave-assisted solvent extraction (MASE) offers significant promise. Microwaves induce rapid heating and pressure accumulation within plant cells, leading to cell wall disruption and enhanced mass transfer [19]. Compared with conventional techniques such as hot water extraction, water–ethanol precipitation, and Soxhlet extraction, MASE provides higher selectivity, shorter extraction time, reduced solvent consumption, and lower energy input, while maintaining the structural integrity of bioactive compounds [20]. The selectivity of microwave-assisted solvent extraction (MASE) stems from two main mechanisms: the differential interaction of microwaves with various substances, and the controlled selection of extraction parameters. The combination of these two factors allows MASE to selectively extract target compounds while minimizing the dissolution of coexisting interferents [20]. Moreover, it is considered a green and sustainable technology with minimal environmental burden [21]. However, since efficient microwave absorption requires polar solvents, the choice of extraction medium remains a key limitation [22]. Despite this, previous studies have demonstrated that MASE can not only improve saponin yield and purity but also drastically shorten processing time, thus increasing the feasibility of industrial application [23]. Overall, considering the broad spectrum of bioactivities of tea saponins and the drawbacks of conventional extraction methods, developing optimized microwave-assisted solvent extraction processes is of great importance. Such technology is expected to achieve high-quality, cost-effective, and environmentally sustainable production of tea saponins, thereby promoting their utilization in high-value applications across multiple industries.

In this study, *C. oleifera* husks collected from three representative regions in Jiangxi Province (Ganzhou—GZ, Yichun—YC, and Jiujiang—JJ, China) were comprehensively characterized in terms of their physicochemical and structural properties to elucidate regional variations and their potential implications for high-value utilization. Single-factor experiments and response surface methodology (RSM) were subsequently applied to optimize the microwave-assisted solvent extraction of tea saponins, with liquid-to-solid ratio, ethanol concentration, extraction time, and microwave power as the main influencing factors. The extraction yields were validated under optimized conditions to confirm the accuracy of the predictive model. By systematically correlating the physicochemical characteristics of husks with extraction performance, this work provides new insights into the structure–function relationship of *C. oleifera* husks and establishes a scientific basis for the efficient conversion of agricultural by-products into high-value bioactive compounds.

## 2. Materials and Methods

### 2.1. Materials

The *C. oleifera* husks were obtained from three different regions in Jiangxi Province (GZ, YC, and JJ, China) and stored for more than 3 months under conditions of 25 °C, relative humidity below 60%, and good ventilation. Before starting the experiment, the C. oleifera husks were dried in a forced-air drying oven at 75 °C for 24 h. The dried husks were crushed into three distinct particle sizes (40–60 mesh, 60–80 mesh, and 80–100 mesh) by a high-speed multi-functional crusher (CS-700, Haina, China) and then sieved. The chemical reference substances used in this study were tea saponin standard (≥98%) and rutin standard (≥98%), which were purchased from Yuan Ye Biotechnology Co., Ltd. (Shanghai, China). Other reagents included aluminum nitrate (analytical grade) purchased from Maclean Biochemical Technology Co., Ltd. (Shanghai, China), anhydrous ethanol, petroleum ether (boiling range: 60–90 °C), concentrated sulfuric acid, vanillin, and anhydrous sodium carbonate purchased from Xilong Science Co., Ltd. (Shenzhen, China); tannic acid (analytical grade), sodium hydroxide, boric acid, sodium nitrite, Folin–Denis reagent, and sodium bisulfite (analytical grade) purchased from Sinopharm Group Chemical Reagent Co., Ltd. (Beijing, China).

### 2.2. Comprehensive Physicochemical and Structural Characterization of C. oleifera Husks

#### 2.2.1. Composition Analysis of *C. oleifera* Husks

The composition analysis of *C. oleifera* Husks was conducted on the samples of 40–60 mesh. Moisture content was determined according to the direct drying method specified in GB 5009.3-2016 National Food Safety Standard—Determination of Moisture in Foods, and the moisture content extraction yield was calculated as follows:

Moisture content yield (%) = [(Wet weight of *C. oleifera* husks − Dry weight of *C. oleifera* husks)/Wet weight of *C. oleifera* husks] × 100% (1)

Protein content was determined according to the Kjeldahl method specified in GB 5009.5-2016 National Food Safety Standard—Determination of Protein in Foods using a Kjeldahl nitrogen analyzer (K9860, Haineng, China). Fat content was determined according to the Soxhlet extraction method specified in GB 5009.6-2016 National Food Safety Standard—Determination of Fat in Foods. Ash content was determined according to the high-temperature ignition gravimetric method specified in GB 5009.4-2016 National Food Safety Standard—Determination of Ash in Foods using a muffle furnace (YX-1000XB, Yaoxing, China).

Cellulose, hemicellulose, and lignin contents were determined using the Van Soest method [24]. The sample was boiled in a neutral detergent solution to obtain neutral detergent fiber (NDF), which includes cellulose, hemicellulose, lignin, and silicates. The NDF residue was then boiled in an acid detergent solution to obtain acid detergent fiber (ADF), consisting of cellulose, lignin, and silicates. The ADF residue was subsequently treated with 72% sulfuric acid to yield acid detergent lignin (ADL), comprising lignin and silicates. Finally, the ADL residue was ashed. The volatile fraction lost during the ashing process represents the lignin content. The cellulose, hemicellulose, and lignin contents were calculated by subtracting the relevant fiber fractions: hemicellulose = NDF—ADF; cellulose = ADF—ADL; lignin = mass loss during the ashing of ADL.

#### 2.2.2. Elemental Analysis of *C. oleifera* Husks

The carbon (C), hydrogen (H), nitrogen (N), and sulfur (S) contents in *C. oleifera* husk 40–60 mesh powder were determined using an elemental analyzer (Vario EL cube, Elimonta, Germany). Oxygen (O) content was determined indirectly by difference [25].

#### 2.2.3. BET Analysis of *C. oleifera* Husks

The specific surface area and pore size distribution of *C. oleifera* husk powders with particle sizes of 40–60, 60–80, and 80–100 mesh across three regions were analyzed using a fully automated surface area and porosity analyzer (TriStar II 3020, Micromeritics, Norcross, GA, USA), with high-purity nitrogen (99.99%) used as the adsorption medium. Three different particle sizes were selected for BET analysis because particle size can significantly influence the test results. Nitrogen adsorption–desorption isotherms were measured at 77.3 K. The Brunauer–Emmett–Teller (BET) [26] method and the Barrett–Joyner–Halenda (BJH) [27] theory were applied to calculate the specific surface area, mesopore volume, and pore size distribution of the samples.

#### 2.2.4. SEM Analysis of *C. oleifera* Husks

Representative dried *C. oleifera* husk 80–100 mesh powder samples were evenly dispersed on specimen stubs using double-sided adhesive tape. In scanning electron microscopy, smaller particle sizes are selected to improve imaging quality, obtain accurate surface information, and minimize charging effects. After sputter coating with gold, the microstructure of the powders was observed using a field-emission scanning electron microscope (SEM) (JSM-7900F, JEOL, Tokyo, Japan). Images were captured at magnifications of 150× and 500× to examine the micromorphology and surface structure [28].

#### 2.2.5. FTIR Spectroscopy Analysis of *C. oleifera* Husks

A small amount of *C. oleifera* husk powder (80–100 mesh) from three regions was thoroughly mixed and ground with KBr powder. When conducting infrared spectroscopy analysis of a sample, a finer particle size is preferred to minimize light scattering and thereby obtain high-quality spectra with an enhanced signal-to-noise ratio and accurate representation of infrared absorption characteristics. The mixture was pressed into a transparent pellet using a hydraulic press. The sample was introduced into an infrared spectrometer (Nicolet iS5, Nicolet, Madison, WI, USA) for scanning, during which a total of 64 scans were performed. The scanning range was set between 4000 and 500 cm^−1^, with a step size of 2 cm^−1^ [29].

### 2.3. Extraction and Determination of Tea Saponins, Tannins, and Flavonoids

**Figure 1:**
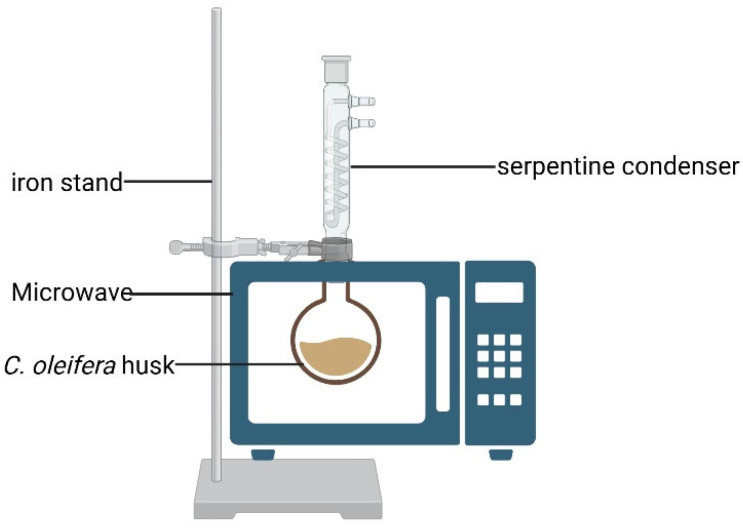
Diagram of the MASE device.

For each extraction, 2 g of powdered *C. oleifera* husks (40–60 mesh) from the GZ, YC, and JJ regions was weighed and placed into a 500 mL round-bottom flask. The mixture was stirred uniformly and then placed into the microwave reactor (NN SM30NW, Panasonic, Xiamen, China). Extraction was conducted at a specified ethanol concentration, solid-to-liquid ratio, microwave power, and extraction time. The ethanol solution that volatilized during the extraction process was completely condensed through a 300 mm long serpentine condenser using water at 0 °C and then refluxed into the reactor. After extraction, vacuum filtration was performed, and the filtrate was diluted to a final volume of 250 mL for subsequent analysis. The Diagram of the MASE device is shown in Figure 1.

#### 2.3.1. Extraction and Determination of Tea Saponins

The concentration of tea saponins in the extract was determined using a spectrophotometer (UV-9000, Yuanxi, China) by the vanillin–concentrated sulfuric acid method [30], with the standard calibration curve given as y = 0.99514x + 0.00158 (R^2^ = 0.9998), where x represents the absorbance at 545 nm of the test solution, and y represents the tea saponin concentration in the test solution. The tea saponin extraction yield was calculated as follows:

Tea saponin extraction yield (%) = [Amount of tea saponin in extract solution/Dry weight of *C. oleifera* husks] × 100% (2)

#### 2.3.2. Extraction and Determination of Tannins

The concentration of tannins in the extract was determined using a spectrophotometer (UV-9000, Yuanxi, China) by the Folin–Ciocalteu method [31], with the standard calibration curve given as y = 1.29932x + 0.00738 (R^2^ = 0.9983), where x represents the absorbance at 760 nm of the test solution, and y represents the tannic acid concentration in the test solution. The tannin extraction yield was calculated as follows:

Tannin extraction yield (%) = [Amount of tannic acid in extract solution/Dry weight of *C. oleifera* husks] × 100% (3)

#### 2.3.3. Extraction and Determination of Flavonoids

The concentration of flavonoids in the extract was determined using a spectrophotometer (UV-9000, Yuanxi, China) following the method of Gudej et al. [32], with the standard calibration curve given as y = 0.6369x + 0.00793 (R^2^ = 0.9984), where x represents the absorbance at 510 nm of the test solution, and y represents the flavonoid equivalent concentration (mg/mL) in the test solution. The flavonoid extraction yield was calculated as follows:

Flavonoid extraction yield (%) = [Amount of flavonoids (as rutin equivalent) in extract solution/Dry weight of *C. oleifera* husks] × 100% (4)

### 2.4. Single-Factor Experiment

Single-factor experiments were conducted to investigate the effects of the solid-to-liquid ratio, ethanol concentration, extraction time, and microwave power on the yields of tannins, flavonoids, and tea saponins [33].

The influence of different solid-to-liquid ratios (1:10, 1:20, 1:40, 1:60 g/mL) on the yield of saponins, tannins, and flavonoids from *C. oleifera* husks across three regions was investigated under fixed conditions: ethanol concentration of 20%, microwave time of 2 min, and microwave power of 350 W.

The influence of different ethanol concentrations (20%, 40%, 60%, 80%) on the yield of saponins, tannins, and flavonoids from *C. oleifera* husks across three regions was investigated under fixed conditions: solid-to-liquid ratio of 1:40 g/mL, microwave time of 2 min, and microwave power of 350 W.

The influence of different microwave extraction times (2, 4, 6, 8 min) on the yield of saponins, tannins, and flavonoids from *C. oleifera* husks across three regions was investigated under fixed conditions: solid-to-liquid ratio of 1:40 g/mL, ethanol concentration of 40%, and microwave power of 350 W.

The influence of different microwave power levels (70, 210, 350, 490 W) on the yield of saponins, tannins, and flavonoids from *C. oleifera* husks across three regions was investigated under fixed conditions: solid-to-liquid ratio of 1:40 g/mL, ethanol concentration of 40%, and microwave time of 2 min.

### 2.5. Response Surface Experiment

Based on the results of the single-factor experiment involving tea saponin, tannin, and flavonoid extraction yields, four key parameters—liquid-to-solid ratio (A), ethanol concentration (B), microwave extraction time (C), and microwave power (D)—were selected for further investigation [33]. Using the extraction yield of tea saponin as the response variable, response surface methodology (RSM) experiments were conducted on *C. oleifera* husk samples of 40–60 mesh collected from three different regions. A four-factor, three-level experimental design was developed using the Box–Behnken design principle in Design Expert 8.0 software (Table 1).

### 2.6. Statistical Analysis

All the experiments were conducted three times, and the results were presented as mean ± standard deviation (SD). Statistical significance analysis, including one-way analysis of variance (ANOVA) and correlation analysis combined with Tukey’s HSD post hoc test, was performed using IBM SPSS Statistics 27. A *p*-value less than 0.05 was considered statistically significant at the 95% confidence level. Graphs were generated using Origin 2021 software.

## 3. Results and Discussion

### 3.1. Physical and Chemical Properties of C. oleifera Husks in Three Regions

**Figure 2:**
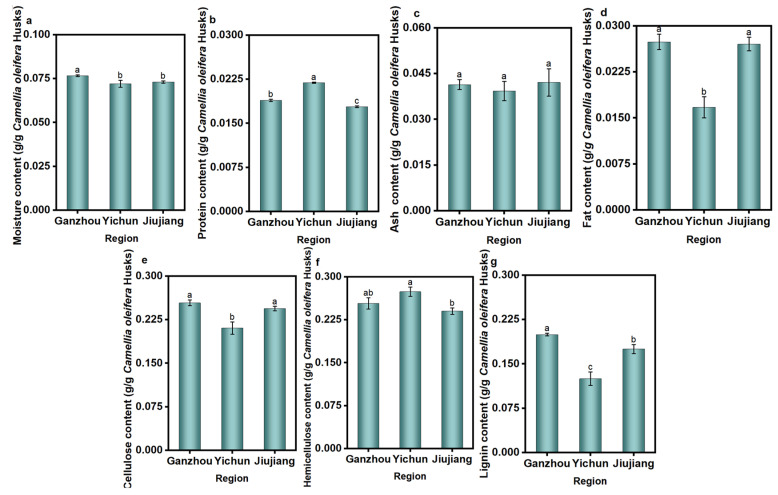
Physical and chemical indicators of *C. oleifera* husks in three regions. (**a**) Moisture; (**b**) protein; (**c**) ash; (**d**) fat; (**e**) cellulose; (**f**) hemicellulose; (**g**) lignin. Different letters (a–c) represent significant differences (*p* < 0.05).

Figure 2 reveals significant regional variations in the composition of *C. oleifera* husks. GZ exhibited the highest moisture content (7.66 ± 0.05%), followed by JJ (7.30 ± 0.07%), with YC having the lowest (7.20 ± 0.20%) (Figure 2a). This variation primarily reflects local climatic conditions during harvest (rainfall, air humidity). JJ’s relatively humid and rainy climate likely contributes to its higher natural moisture content. YC had the highest protein content (2.19 ± 0.01%), surpassing GZ (1.89 ± 0.02%) and JJ (1.78 ± 0.01%) (Figure 2b). This suggests richer soil nitrogen levels in YC, potentially due to higher organic matter content or sufficient fertilization. YC showed the lowest ash content (3.93 ± 0.32%), while JJ had the highest (4.21 ± 0.45%), followed by GZ (4.14 ± 0.17%). However, there was no significant difference in ash content among the three regions (Figure 2c). Although YC’s soil fertility appears comparatively higher (inferred from protein), its lower ash content may indicate reduced absorption capacity for mineral elements by the YC *C. oleifera*. Fat content in YC (1.67 ± 0.17%) was significantly lower than in GZ (2.74 ± 0.13%) and JJ (2.70 ± 0.11%) (Figure 2d). This disparity may stem from genetic differences between regional varieties or provenances of *C. oleifera*, influencing their metabolic pathways and lipid synthesis efficiency in the husks [34]. Ions in ash (such as K^+^, Ca^2+^, Mg^2+^, etc.) can conduct electricity under microwave irradiation, generating electric currents and Joule heat. This complicates the microwave heating behavior: while it may lead to a faster heating rate, it can also cause localized overheating, potentially denaturing or degrading heat-sensitive components [35,36]. Additionally, fats and proteins may interact with microwaves and compete with tea saponin for solvent access, thereby affecting extraction efficiency and purity [37].

The husks in the three regions exhibited distinct fiber compositions (Figure 2e–g). YC contained the highest hemicellulose (27.40 ± 0.80%) along with relatively high cellulose (21.05 ± 1.05%) and lignin (12.48 ± 1.14%) contents. GZ showed the highest cellulose (25.38 ± 0.49%) and lignin (19.96 ± 0.24%) levels, while hemicellulose content (25.36 ± 0.99%) ranked second only to YC. JJ husks had moderate cellulose (24.41 ± 0.38%) and lignin (17.50 ± 0.75%) contents, but the lowest hemicellulose level (24.00 ± 0.56%). The consistently high levels of lignin, cellulose, and hemicellulose indicate strong potential for these husks to serve as biomass energy feedstocks or raw materials for bio-based products. In particular, the elevated lignin content is favorable for enhancing both calorific value and structural hardness [38]. The higher the content of cellulose and lignin, the denser and stronger the cell wall becomes. The microwave extraction method requires higher temperatures and longer processing times to effectively disrupt this structural barrier [39]. Variations in the proportions and structural arrangements of these three components within the cell walls of *C. oleifera* husks across the three regions result in differing microwave energy absorption efficiencies. Consequently, this influences the overall heating rate and the effectiveness of cell wall disruption and by-product extraction [40].

The elemental composition of husks from the three regions showed no significant differences (Table 2). Carbon, the core element constituting the primary components of C. oleifera husks such as lignin, cellulose, and hemicellulose, forms carbon atom networks within these macromolecular organic compounds that serve as excellent microwave absorbers. Oxygen is predominantly present in the glycosidic bonds and hydroxyl groups of cellulose, hemicellulose, and tea saponin molecules, which facilitates penetration and wetting by polar extraction solvents such as ethanol–water solutions. Hydrogen content is closely correlated with moisture content. As a crucial medium in MASE, water molecules—being highly polar—rotate and frictionally interact at high speeds under microwave irradiation, generating substantial heat. This leads to a rapid increase in intracellular pressure, effectively disrupting the cell walls and creating pathways for tea saponin release. Nitrogen, an indicator element for proteins, may undergo denaturation under microwave heating and interact with tea saponin, thereby influencing its dissolution and subsequent separation and purification. Sulfur content likely originates from trace sulfur-containing amino acids or other organic sulfur compounds. While sulfur itself contributes minimally to microwave absorption, it may act as an impurity affecting the purity and color of the final tea saponin product [8,21,41].

### 3.2. Structural Characterization of C. oleifera Husks in Three Regions

#### 3.2.1. BET Analysis of *C. oleifera* Husks

**Figure 3:**
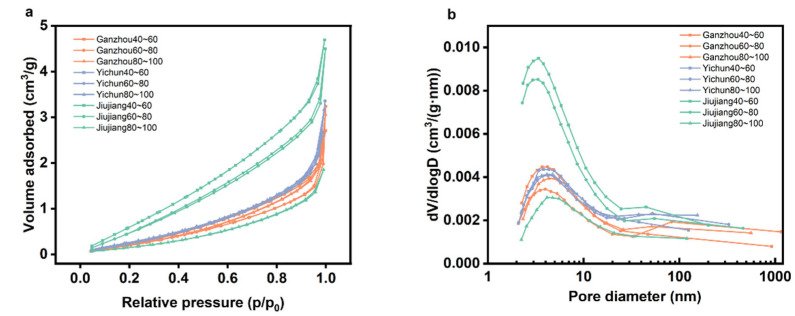
The nitrogen adsorption and desorption curves and pore size distribution curves of *C. oleifera* husks in three regions. (**a**) Nitrogen adsorption and desorption curves; (**b**) pore size distribution curves.

According to the International Union of Pure and Applied Chemistry (IUPAC) classification of adsorption–desorption isotherms [42], the nitrogen adsorption–desorption isotherms shown in Figure 3 for *C. oleifera* husks correspond to Type III. Type III isotherms typically show no significant hysteresis loop. The desorption branch closely follows or lies directly on top of the adsorption branch, indicating reversibility and the absence of delayed desorption caused by pore confinement effects (e.g., capillary condensation). This further supports the material’s lack of significant mesoporous or microporous structure (or, if pores are present, very weak interactions between pore walls and N_2_).

There is a positive correlation between the particle size of oil tea seed husks and their BET surface area. Larger particles generally exhibit a larger BSA. Under the same particle size range, significant differences in BET surface area were observed among husks from JJ, GZ, and YC. Specifically, JJ samples showed a higher BET surface area in the 40–60 and 60–80 mesh fractions compared to those from GZ and YC, but a lower BSA in the 80–100 mesh fraction (Table 3). A larger BET surface area typically enhances the rate of MASE by providing more sites for microwave interaction, thereby improving the conversion of microwave energy into thermal energy. It also facilitates greater solvent accessibility through increased contact and penetration pathways, promoting faster dissolution of solutes such as tea saponin, tannins, and flavonoids [43]. Similarly, a larger pore volume allows more solvent to be retained, enhancing mass transfer between solid and liquid phases, while also reducing diffusion resistance within particles, thereby aiding the outward transport of solute molecules [44]. In the 40–60 and 60–80 mesh fractions, the average pore size followed the order: YC > GZ > JJ. In the 80–100 mesh fraction, the order was GZ > JJ > YC. However, the most probable pore size showed little variation among the three regions and fell consistently within the micropore range (<2 nm) (Table 3). Elemental analysis revealed that *C. oleifera* husks exhibit high carbon content along with a distinct pore structure, making them a high-quality raw material for activated carbon preparation. The sample from the JJ region, which contains larger pores, demonstrates a relatively higher specific surface area and consequently leads to activated carbon with superior adsorption performance [7].

#### 3.2.2. SEM Analysis of *C. oleifera* Husks

**Figure 4:**
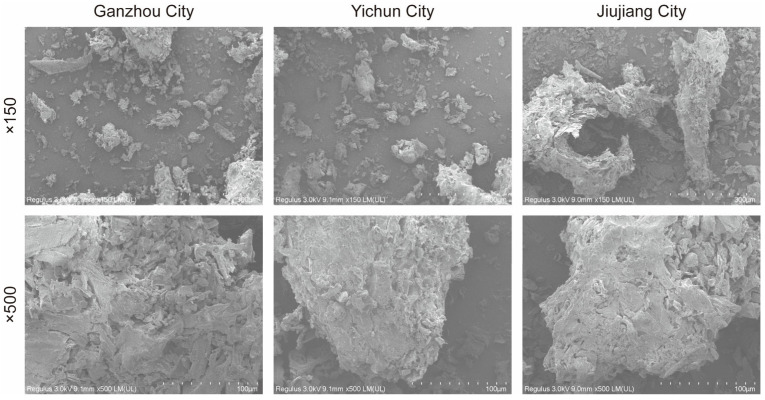
The scanning electron microscopy images of 80–100 mesh *C. oleifera* husks in three regions.

The SEM Analysis of *C. oleifera* Husks are shown in Figure 4. At 150-times magnification, *C. oleifera* husk powder exhibits irregularly shaped, cavity-containing block structures with non-uniform particle sizes. Its surface appears porous and uneven, with surface microstructures remaining indistinct. At 500-times magnification, according to the scale bar in the images, the aperture should be less than 1 μm. Combined with BET analysis, the results indicate that the majority of the pores in *C. oleifera* husks from the three regions are around 2 nm in size. The pores observed in the SEM images may represent larger pores or clustered arrangements of smaller pores. If further magnified to the nanometer scale, a loose, honeycomb-like porous network structure would likely be revealed. The surface is rough and uneven, yet the spatial architecture shows relative uniformity [45]. This honeycombed microporosity significantly increases the powder’s surface area, enhancing its hydrophilic properties and resulting in superior water- and oil-holding capacities.

The pores and cavities on the surface of *C. oleifera* husks serve as natural pathways for solvent penetration and the release of intracellular compounds. A higher porosity and a greater number of channels increase the specific surface area of the raw material, which reduces mass transfer resistance and enhances the contact area between solute and solvent, thereby facilitating extraction.

#### 3.2.3. FTIR Spectrum Analysis of *C. oleifera* Husks

**Figure 5:**
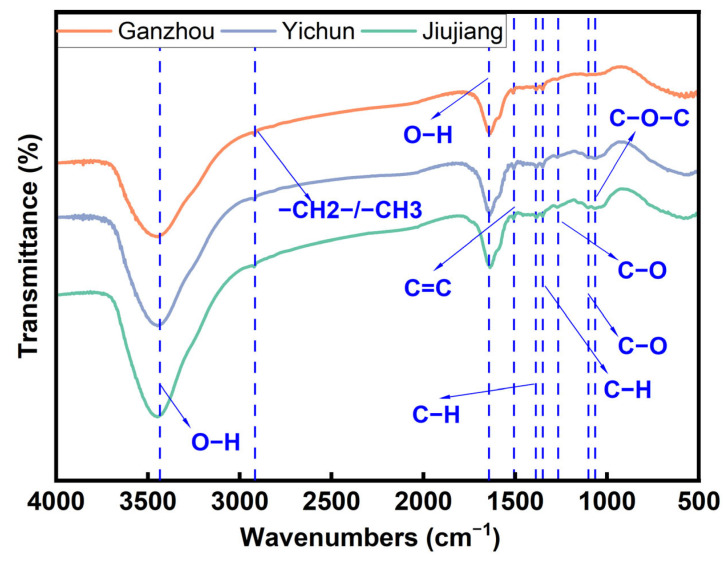
Infrared spectra of *Camellia oleifera* in three regions.

The FITR spectra of *C. oleifera* husks (Figure 5) from three regions exhibit broadly similar peak profiles, indicating no significant differences in their primary functional groups. At ~3435 cm^−1^ [46], a strong, broad absorption peak is observed, attributed to O−H stretching vibrations of both aliphatic and aromatic hydroxyl groups. Notably, the JJ sample exhibits a more intense peak here than the other two regions, suggesting a higher hydroxyl group content. At 2927 cm^−1^, this peak corresponds to C−H stretching vibrations of methylene (−CH_2_−) and methyl (−CH_3_) groups. At 1632 cm^−1^, the absorption arises from O−H bending vibrations. At 1510 cm^−1^, the peak signifies aromatic ring skeletal vibrations (C=C), a characteristic absorption band for lignin in *C. oleifera* husks. The peaks at 1384 cm^−1^ and 1350 cm^−1^ are primarily associated with C−H bending vibrations of methyl groups (−CH_3_) and O−H in-plane bending vibrations [46]. At 1268 cm^−1^, the absorption is assigned to aromatic C−O stretching vibrations and O−H in-plane bending vibrations. This is a characteristic peak for guaiacyl units in lignin, often correlated with aromatic ring C−O stretching, serving as another key indicator of lignin [39]. At 1102 cm^−1^, the peak mainly arises from aliphatic C−O stretching vibrations. Finally, at 1062 cm^−1^, the absorption corresponds to asymmetric C−O−C stretching vibrations [46]. The *C. oleifera* husks from three different regions contain both hydrophilic groups (such as hydroxyl groups) and hydrophobic components (like lignin). Therefore, in MASE, an ethanol–water mixture is often the preferred solvent [6]. The spectral peaks corresponding to characteristic functional groups in tea saponin molecules, such as −OH, C−H, C−O, and glycosidic bonds, showed variations in intensity among the three regional samples of *C. oleifera* husks. These spectral differences suggest potential variations in tea saponin content [12].

### 3.3. Effect of Extraction Parameters on the Yield of Tea Saponins

**Figure 6:**
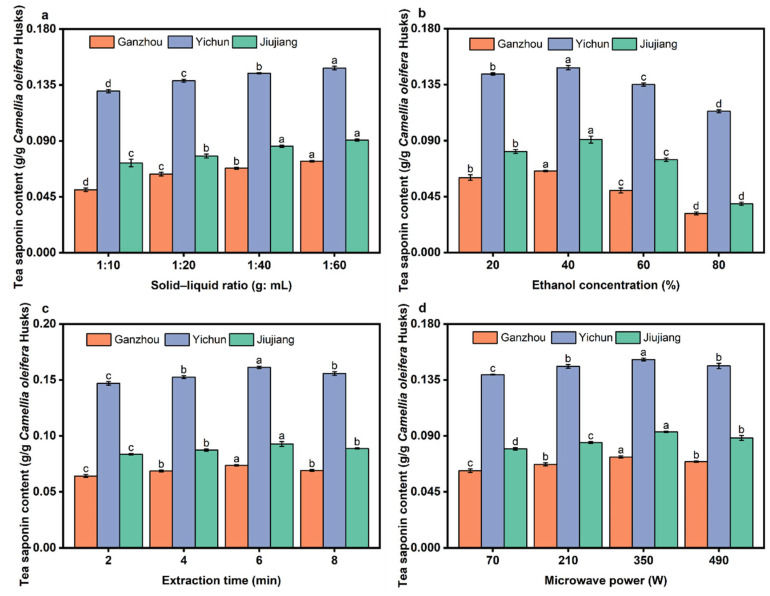
The effect of extraction parameters on the yield of tea saponins. (**a**) Solid–liquid ratio; (**b**) ethanol concentration; (**c**) microwave extraction time; (**d**) microwave power. Different letters (a–d) represent significant differences (*p* < 0.05).

Under fixed conditions—ethanol concentration of 20%, microwave time of 2 min, and microwave power of 350 W—as the solid-to-liquid ratio increased from 1:10 to 1:40, the yield of tea saponins consistently rose (Figure 6a). This improvement is attributed to insufficient solvent volume at lower ratios (e.g., 1:10), which fails to fully penetrate all raw materials. Consequently, some tea saponins in the *C. oleifera* husk cannot effectively contact and dissolve in the ethanol solvent. Additionally, the low ratio results in a highly viscous system, increasing resistance to solvent diffusion and hindering the mass transfer of tea saponin from the husk interior to the bulk solvent, thereby reducing the extraction rate. Moreover, the thickened mixture may lead to uneven microwave heating, creating localized hot or cold spots that compromise overall extraction efficiency. When the ratio increased from 1:40 to 1:60, the yield showed a tendency to increase, but the increase rate plateaued. Simultaneously, the microwave energy absorbed per unit volume of solvent decreases, slowing the overall heating rate or preventing the system from reaching the target temperature. This weakens the thermal effect of microwaves and their ability to disrupt cell walls [20]. Considering economic efficiency, an optimal solid-to-liquid ratio of 1:40 is recommended.

Extraction results across the three regions demonstrate similar trends in tea saponin yield relative to ethanol concentration. Under fixed conditions—solid-to-liquid ratio of 1:40 g/mL, microwave time of 2 min, and microwave power of 350 W—as the ethanol concentration increased from 20% to 40% (*v*/*v*), the yield of tea saponins significantly increased (Figure 6b). The addition of ethanol reduces solvent polarity, markedly enhancing the solubility of tea saponin. Ethanol disrupts the strong hydrogen-bonding network between water molecules, lowers solution surface tension, and improves penetration into plant cells. This enhances the dissolution of tea saponin, particularly its glycoside moieties and hydrophobic structures. At 40% ethanol (*v*/*v*), the solvent polarity is optimal, effectively dissolving both hydrophilic sugar groups and hydrophobic aglycone parts, resulting in the highest extraction efficiency. However, when ethanol concentration increased from 40% to 60% (*v*/*v*), the extraction yield decreased. Tea saponin contains multiple hydrophilic hydroxyl groups and sugar chains; excessively high ethanol concentrations may reduce its solubility, potentially causing partial precipitation or incomplete dissolution. Furthermore, the dissolution of water-soluble impurities (e.g., proteins, sugars, amino acids, tannins) increases. These impurities can form precipitates with tea saponin, negatively impacting the extraction yield via MASE.

Under fixed conditions—solid-to-liquid ratio of 1:40 g/mL, ethanol concentration of 40%, and microwave power of 350 W—as the extraction time increased from 2 to 6 min, the yield of tea saponins continuously increased (Figure 6c). Shorter times (e.g., 2 min) provide insufficient microwave energy input, leading to inadequate cell wall disruption and leaving most tea saponins trapped within the raw material, resulting in low yield. The maintained concentration gradient, combined with thermal effects and high-speed molecular motion, synergistically disrupts cell structures, facilitating the rapid release of tea saponin from the cells. Beyond 4–6 min of extraction, the yield of tea saponins began to decline. Most readily extractable tea saponins had already been dissolved. Concurrently, prolonged microwave irradiation may cause thermal degradation, oxidation, or structural changes (e.g., deglycosylation, ring cleavage) of the released tea saponins.

Under fixed conditions—solid-to-liquid ratio of 1:40 g/mL, ethanol concentration of 40%, and microwave time of 2 min—as microwave power increased from 70W to 350 W (Figure 6d), the yield of tea saponins significantly increased. Insufficient power (e.g., 70 W) results in slow heating, selectively dissolving easily released components with minimal impurity co-extraction. Higher power accelerates the heating rate, enabling the system to rapidly reach the temperature (generally 60 °C) required for efficient tea saponin extraction. This temperature is crucial for disrupting cell walls, reducing solvent viscosity, and accelerating diffusion [47]. Even at the lowest power setting, the temperature measured after extraction reached 80 °C. The temperature variation after microwave extraction under different experimental conditions was not significant, ranging from 80 to 85 °C. Moreover, vigorous boiling of the solution was clearly observed during microwave heating, demonstrating the highly efficient and rapid nature of microwave-assisted heating. The maximum yield of tea saponins occurred at 350 W. Further increasing power to 490 W led to a decrease in yield. Tea saponin, being a saponin compound, is relatively heat-sensitive. Excessive power causes a rapid, uncontrollable temperature rise and exacerbates uneven heating within the system. This can damage the chemical structure of tea saponin (e.g., glycosidic bond cleavage, aglycone decomposition, dehydration), leading to its decomposition or conversion into other substances [33]. Additionally, excessive power causes rapid boiling and excessive solvent evaporation. This prevents adequate contact between the husk and the solvent, and alters the extraction environment, further reducing the yield of tea saponins [48].

Significant differences were observed in the tea saponin content of *C. oleifera* husks among the three regions. YC exhibited the highest content, followed by JJ, while GZ showed the lowest. This disparity is likely attributable to the long-term cultivation of distinct *C. oleifera* husk varieties in each region. Furthermore, YC’s soil structure, fertility, and climatic conditions appear to be more conducive to the metabolic synthesis of tea saponin in the *C. oleifera* husk. As the traditional core production area in Jiangxi, YC may also benefit from superior cultivars and more refined cultivation practices, collectively contributing to a higher and more balanced quality of bioactive constituents [34,49]. This study found that the saponin content in the YC area was higher than the 11.53% reported in the literature for *Swietenia mahogany* Jacq [50], while the contents in the GZ and JJ areas were lower.

### 3.4. Effect of Extraction Parameters on the Yield of Tannins

**Figure 7:**
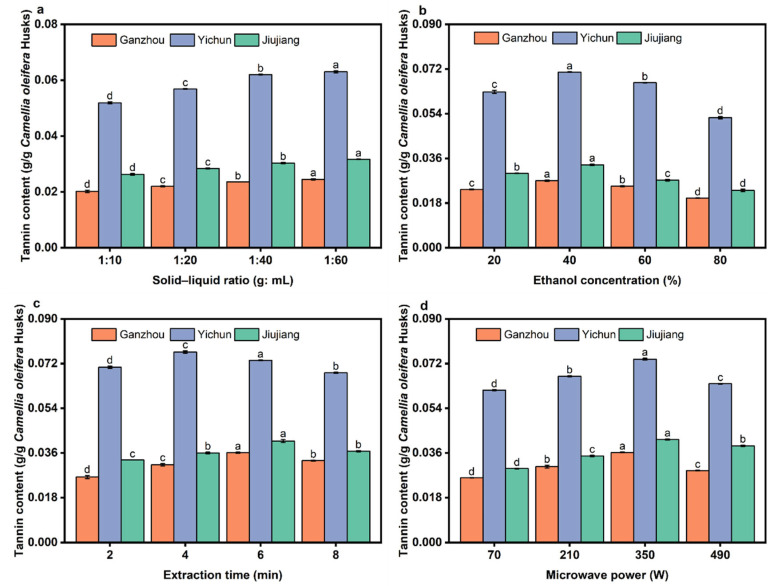
The effect of extraction parameters on the yield of tannins. (**a**) Solid–liquid ratio; (**b**) ethanol concentration; (**c**) microwave extraction time; (**d**) microwave power. Different letters (a–d) represent significant differences (*p* < 0.05).

Figure 7a shows that the variation trend of tannin extraction yield with the solid-to-liquid ratio is generally similar across the three regions. As the ratio increases from 1:10 to 1:40, the solvent adequately wets the *C. oleifera* husks, ensuring uniform microwave energy absorption by the material. This promotes cell wall rupture and component release. The ratio of 1:40 is optimal for the yield of tannins. When the ratio increases to 1:60, the yield shows a tendency to increase, but the increase rate plateaus. This also leads to increased energy consumption, solvent waste, and substantially higher subsequent processing costs, making it economically inefficient [51].

The highest tannin yield was achieved at 40% ethanol concentration (Figure 7b). When the ethanol concentration increased further, the solubility of tannins decreased significantly due to their polyphenolic hydroxyl groups, which make them more hydrophilic.

Figure 7c reveals regional differences in the yield of tannins with different microwave extraction times. The yield in YC peaks at 4 min, while yields in GZ and JJ both peak at 6 min. The *C. oleifera* husks from the YC region exhibit the lowest cellulose and lignin contents among the compared areas. As the major structural components of the cell walls, cellulose and lignin represent the main obstacles during extraction processes. Cellulose, which forms the primary supporting framework of the cell wall, can inhibit the release of tannins [23]. Lignin acts as a binder for cellulose and hinders the penetration of solvents. Compared with the other two regions, the *C. oleifera* husks from YC likely contain relatively higher amounts of free-form tannins, which can be rapidly released from ruptured cells in a short time under microwave treatment [52].

As the power increases from 70 W to 350 W, the yield of tannins rises significantly (Figure 7d). Higher power enables rapid and efficient cell wall disruption, accelerating tannin diffusion from the raw material into the solvent. The peak tannin yield is achieved at 350 W. Further power increase causes water vaporization within the system, leading to a sharp rise in internal pressure and localized overheating. This results in tannin degradation. The tannin content obtained in this study from all three regions was lower than the literature value for *Pithecellobium jiringa* (11.53%) [53], demonstrating that *C. oleifera* husks are not a rich source of tannins.

### 3.5. Effect of Extraction Parameters on the Yield of Flavonoids

**Figure 8:**
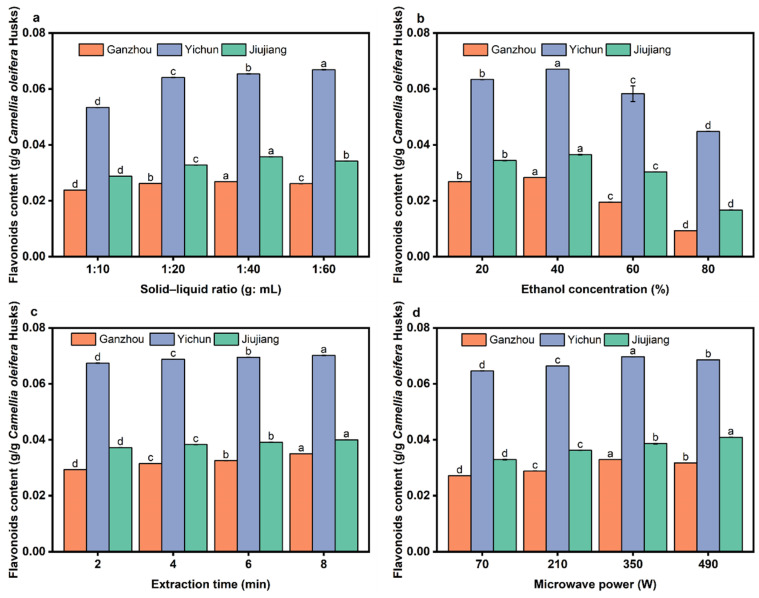
The effect of extraction parameters on the yield of flavonoids. (**a**) Solid–liquid ratio; (**b**) ethanol concentration; (**c**) microwave extraction time; (**d**) microwave power. Different letters (a–d) represent significant differences (*p* < 0.05).

As shown in Figure 8, the trends of flavonoid extraction yield versus solid-to-liquid ratio are generally similar across the three regions. As the ratio increased from 1:10 to 1:40, the solvent gradually became sufficient to fully infiltrate the raw material, allowing more flavonoids to dissolve (Figure 8a). At a ratio of 1:40, flavonoids dissolved adequately with minimal mass transfer resistance, resulting in the peak extraction yield. Further increasing the ratio to 1:60 slightly improved the extraction yield. However, it significantly decreased the flavonoid concentration per unit volume of solvent [54].

At 20% ethanol concentration, the polarity is close to that of water, resulting in poor solubility for medium-to-low polarity flavonoids (e.g., flavonoid aglycones) and a low extraction yield (Figure 8b). At 40% ethanol, the polarity and hydrogen-bonding capacity were balanced, enabling high solubility for most flavonoids (both aglycones and glycosides). This concentration also offered suitable polarity and microwave responsiveness, facilitating efficient energy transfer and target compound dissolution. At 60% and 80% ethanol, the increased lipophilicity and reduced hydrogen-bonding weakened the extraction efficiency for polar flavonoids (e.g., glycosides).

As can be seen in Figure 8c, the trends of flavonoid extraction yield versus microwave extraction time are not pronounced across the three regions. Although the yield increased slightly with longer extraction times, the growth rate plateaued. This is because, under these conditions, microwaves cause water vaporization within *C. oleifera* husk cells, generating high pressure that rapidly releases flavonoids within 1–3 min. Prolonged microwave heating will promote flavonoid oxidation, hydrolysis, or isomerization [54].

Figure 8d shows the regional variations in flavonoid extraction yield versus microwave power. The yields for GZ and YC peaked at 350 W, while JJ exhibited a slight further increase even at 490 W. The *C. oleifera* husks from JJ exhibit a larger specific surface area compared to those from GZ and YC. However, due to their relatively smaller pore size, it is difficult for large molecules such as flavonoids to penetrate. The contents of the three major structural components (cellulose, hemicellulose, and lignin) in JJ samples are slightly lower than those in GZ but higher than in YC. Meanwhile, the flavonoid content in JJ samples is higher than that in GZ, which necessitates the use of higher microwave power to achieve complete flavonoid release. Additionally, the relatively high oil content in JJ *C. oleifera* husks reduces the overall microwave absorption capacity of the material, further increasing the required microwave power for effective extraction [55]. The maximum flavonoid extraction yield obtained in this study from all three regions was higher than the literature value for *Eucommia ulmoides* leaves (2.454%) [56], demonstrating that *Camellia oleifera* husk is a good source of flavonoid compounds. Flavonoids have effects in cardiovascular and anti-cancer aspects [57], so the *C. oleifera* husks can be used in the development of medicinal and health products.

### 3.6. Analysis of Response Surface Optimization Experimental Results

#### 3.6.1. Response Surface Optimization of Tea Saponin Extraction in GZ Region

Based on the results of the single-factor experiment on tea saponins in GZ, the effects of liquid-to-solid ratio (A), ethanol concentration (B), microwave extraction time (C), and microwave power (D) on the extraction yield of tea saponins were investigated, with the extraction yield of tea saponins as the response value. The experimental design and results are shown in Table 4.

The extraction yields of tea saponins obtained under different experimental conditions in Table 4 were fitted by using Design Expert 8.0 software, and a quadratic multiple regression model was obtained:

Extraction yield of tea saponins (%) =  +7.30 + 0.36 × A − 0.51 × B + 0.045 × C
+ 0.12 × D + 0.015 × AB − 0.03 × AC + 0.037 × AD − 0.012 × BC − 5 × 10^−3
^ × BD − 0.018 × CD − 0.075 × A^2^ − 1.03 × B^2^
− 0.53 × C^2^ − 0.48 × D^2^

Analysis of variance was conducted on the regression model, and the results are shown in Table 5

As shown in Table 5, the model yields an F-value of 211.04 with a significance level of *p* < 0.0001, indicating a highly significant model that effectively captures the relationship between the response variable and the influencing factors. The coefficient of determination (R^2^) for the model is 0.9960, suggesting a strong goodness of fit, with 99.60% of the variation in the experimental data being explained by the model. The adjusted R^2^ value is 0.9912, demonstrating that the model accounts for a substantial proportion of variance in the response with respect to the independent variables—tea saponins, liquid-to-solid ratio, ethanol concentration, extraction time, and microwave power. These variables collectively explain 99.12% of the variation in the extraction efficiency. The coefficient of variation (CV) is 0.98%, indicating a high degree of experimental stability and reliability. Additionally, the lack-of-fit F-value is 0.12, with a corresponding *p*-value of 0.9931 (>0.05), which confirms that the lack-of-fit term is not statistically significant. This further supports the conclusion that the model fits the data well and possesses high predictive accuracy [43].

According to the F-values and significance levels (*p*-values) presented in Table 4, the effects of factors A, B, and D on the response variable are highly statistically significant. This indicates that the liquid-to-solid ratio, ethanol concentration, and microwave power exert a highly significant influence on the extraction rate of tea saponins. Additionally, factor C (extraction time), with a *p*-value less than 0.05, demonstrates a statistically significant effect on the extraction efficiency. Based on the magnitude of the F-values, the order of influence on the extraction rate of tea saponins is as follows: ethanol concentration > liquid-to-solid ratio > microwave power > extraction time [55]. The ethanol concentration exerts the most pronounced effect, as it directly determines the solubility and partition coefficient of tea saponin in the solvent. Any deviation from the optimum significantly reduces extraction efficiency. The liquid-to-solid ratio ranks second in influence; with ethanol concentration fixed, this parameter shifts the extraction equilibrium, though benefits diminish once solvent sufficiency is reached. Microwave power has the third greatest impact, as even the lowest applied setting effectively disrupted cell structures, with higher levels yielding only marginal improvements. Extraction time showed the least effect, indicating that under the combined influence of other optimized conditions, the process proceeds rapidly and efficiently, making time a non-limiting factor. [58].

**Figure 9:**
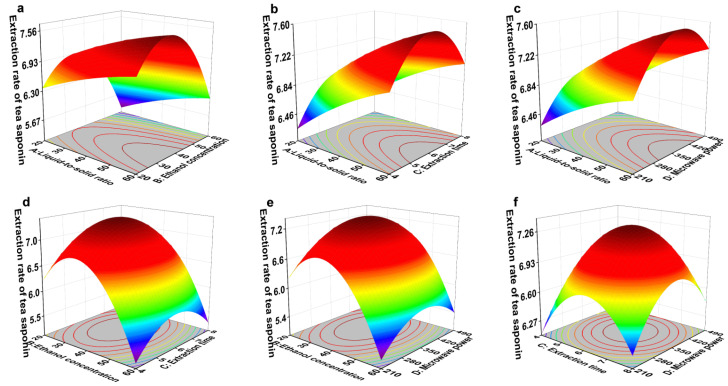
Response surface graph of the effects of liquid-to-solid ratio (A), ethanol concentration (B), extraction time (C), and microwave power (D) on tea saponin extraction yield from *C. oleifera* husks in the GZ region. (**a**) AB; (**b**) AC; (**c**) AD; (**d**) BC; (**e**) BD; (**f**) CD. The peaks of the surface are usually colored yellow/red to indicate a high response value. The low-lying areas of the curved surface are usually colored blue/green to indicate a low response value.

The response surface plot for the yield of tea saponins is shown in Figure 9. Steeper response surfaces denote more significant interactions between factors. As indicated in Table 5, all interaction terms (*p*-value > 0.05 for partial regression coefficients) indicate that no pairwise interactions exhibited statistically significant effects on the yield of tea saponins [59].

The extraction yield of tea saponins was optimized using response surface methodology (RSM). The theoretically optimal extraction conditions were determined as follows: ethanol concentration of 35.5%, liquid-to-solid ratio of 46.75, microwave power of 397.25 W, and extraction time of 5.92 min. Under these conditions, the predicted tea saponin extraction yield was 7.46%.

To verify the reliability of the theoretical optimum, practical extraction conditions were adjusted for operational convenience: liquid-to-solid ratio of 46.75, ethanol concentration of 35.5%, extraction time of 6 min, and microwave power of 350 W. Validation experiments conducted under these modified conditions yielded an actual tea saponin extraction rate of 7.49 ± 0.01%, representing a relative error of 0.38% compared to the theoretical prediction. These results confirm the model’s validity in representing the actual extraction process [60].

#### 3.6.2. Response Surface Optimization of Tea Saponin Extraction in YC Region

Based on the results of the single-factor experiment on tea saponins in YC, the effects of liquid-to-solid ratio (A), ethanol concentration (B), microwave extraction time (C), and microwave power (D) on the extraction yield of tea saponins were investigated, with the extraction yield of tea saponins as the response value. The experimental set and results are shown in Table 6.

The extraction yields of tea saponins obtained under different experimental conditions in Table 6 were fitted using Design Expert 8.0 software, and a quadratic multiple regression model was obtained:

Extraction yield of tea saponins (%) =

+ 16.13 + 0.34 × A − 0.21 × B + 0.21 × C + 0.22 × D + 0.43 × AB + 0.025 × AC + 0.20 × AD − 0.065 × BC − 0.15 × BD + 0.050 × CD − 0.23 × A^2^ − 1.16 × B^2^ − 0.71 × C^2^ − 0.59 × D^2^

Analysis of variance was conducted on the regression model, and the results are shown in Table 7.

As shown in Table 7, the model yielded an F-value of 74.09 with a significance level of *p* < 0.0001, indicating that the model is highly significant and effectively captures the relationship between the response variable and the factors. This demonstrates strong statistical relevance. The coefficient of determination (R^2^ = 0.9886) indicates excellent model fit, explaining 98.86% of the experimental data variability. The adjusted R^2^ value of 0.9752 confirms a significant linear relationship between tea saponin yield and the four factors (liquid-to-solid ratio, ethanol concentration, extraction time, and microwave power), accounting for 97.52% of the variation in extraction yield. The low coefficient of variation (CV = 0.73%) reflects good experimental reliability. Furthermore, the non-significant lack-of-fit term (F = 0.32, P = 0.9109 > 0.05) confirms the model’s adequacy and good predictive accuracy [43].

The F-values and significance levels (*p* < 0.0001) in Table 6 demonstrate that all individual model terms (A, B, C, D) exhibit highly significant effects on the response variable. This confirms that the liquid-to-solid ratio, ethanol concentration, extraction time, and microwave power each exert a highly significant influence on tea saponin extraction yield. Based on their F-values, the relative impact of these factors on the extraction yield follows this order: liquid-to-solid ratio > microwave power > ethanol concentration > extraction time [55]. The tea saponin content in YC is significantly higher than that in GZ. During MASE, this may necessitate more ethanol to reach saturation, making the liquid-to-solid ratio the most influential factor. Microwave power ranks second in terms of impact, which could be attributed to the relatively higher hemicellulose content in the YC samples. This structural characteristic results in denser *C. oleifera* husk tissues that can absorb and dissipate mechanical forces, making the cell walls more resistant to rupture under microwave irradiation. Hence, higher microwave power is required to achieve effective cell wall disruption. As the influence of both the liquid-to-solid ratio and microwave power increases, the relative impact of ethanol concentration decreases accordingly. Once again, extraction time shows the least effect, underscoring the efficiency and rapidity of MASE.

**Figure 10:**
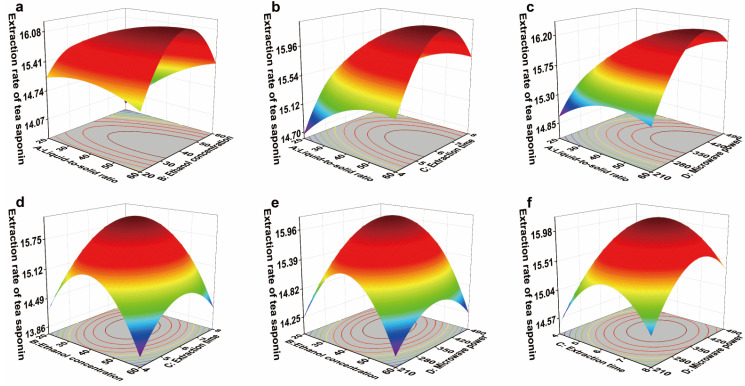
Response surface graph of the effects of liquid-to-solid ratio (A), ethanol concentration (B), extraction time (C), and microwave power (D) on the tea saponin extraction yield from *C. oleifera* husks in the YC region. (**a**) AB; (**b**) AC; (**c**) AD; (**d**) BC; (**e**) BD; (**f**) CD. The peaks of the surface are usually colored yellow/red to indicate a high response value. The low-lying areas of the curved surface are usually colored blue/green to indicate a low response value.

Figure 10 presents the response surface plot for the tea saponin extraction yield in the YC region. A steeper slope (higher inclination) on the 3D surface signifies a more pronounced interaction between factors. Concurrently, an intensifying surface color reflects a sharper increase in the extraction yield [61].

Analysis of the partial regression coefficients in Table 7 reveals that the BD and AD interaction terms were significant (*p* < 0.05). This indicates that the interaction between ethanol concentration and microwave power has a significant effect on tea saponin extraction yield. The dielectric properties of ethanol–water mixtures are not a simple linear superposition but rather undergo complex variations with changing concentration. This implies that different ethanol concentrations create distinct extraction environments with significantly different microwave absorption capacities [62]. The interaction between solid-to-liquid ratio and microwave power also has a highly significant effect on tea saponin extraction yield. Microwave heating is characterized by “volumetric heating” and “selective heating”, with its efficiency being highly dependent on the dielectric properties of the reaction system. The amount of solvent directly influences both the dielectric constant and the total volume of the system. As a result, the optimal values for these parameters are not fixed but rather vary interactively with changes in each other [63].

Optimization of tea saponin extraction yield via response surface methodology (RSM) determined the following theoretical optimal conditions: liquid-to-solid ratio of 50.55, microwave power of 390.60 W, ethanol concentration of 40.13%, and extraction time of 6.36 min. Under these conditions, the predicted extraction yield reached 16.31%.

To validate the theoretical optimum under practical conditions, operational parameters were adjusted as follows: liquid-to-solid ratio of 50.55, ethanol concentration of 40.13%, extraction time of 6 min, and microwave power of 350 W. Validation experiments conducted with these modified parameters yielded an actual tea saponin extraction yield of 16.29 ± 0.02%. This represents a relative error of merely 0.14% compared to the theoretical prediction, confirming the model’s validity in representing the actual extraction process [60].

#### 3.6.3. Response Surface Optimization of Tea Saponin Extraction in the JJ Region

Based on the results of the single-factor experiment on tea saponins in JJ, the effects of liquid-to-solid ratio (A), ethanol concentration (B), microwave extraction time (C), and microwave power (D) on the extraction yield of tea saponins were investigated, with the extraction yield of tea saponins as the response value. The experimental design and results are shown in Table 8.

The extraction yields of tea saponins obtained under different experimental conditions in Table 8 were fitted using Design Expert 8.0 software, and a quadratic multiple regression model was obtained:

Extraction yield of tea saponins (%) =

+9.32 + 0.36 × A − 0.22 × B + 0.13 × C + 0.057 × D − 0.070 × AB − 0.038 × AC + 0.10 × AD + 0.063 × BC − 0.012 × BD − 0.14 × CD − 0.54 × A^2^ − 1.13 × B^2^ − 0.14 × C^2^ − 0.40 × D^2^

Analysis of variance was conducted on the regression model, and the results are shown in Table 9.

Table 9 indicates that the model achieved an exceptionally high F-value of 214.02 (*p* < 0.0001), demonstrating that it is highly significant and effectively captures the relationship between the response variable and the independent factors. The coefficient of determination (R^2^ = 0.9960) signifies an excellent model fit, explaining 99.60% of the experimental data variability. The adjusted R^2^ value of 0.9914 further establishes a highly significant linear relationship between tea saponin yield and the four factors (liquid-to-solid ratio, ethanol concentration, extraction time, and microwave power), accounting for 99.14% of the variation in extraction yield. The remarkably low coefficient of variation (CV = 0.73%) reflects high experimental reliability. Additionally, the non-significant lack of fit (F = 0.61, *p* = 0.7554 > 0.05) confirms the model’s adequacy and high predictive accuracy [43].

Analysis of F-values and significance levels (p) in Table 9 reveals that factors A (liquid-to-solid ratio), B (ethanol concentration), and C (extraction time) exhibit highly significant effects (*p* < 0.0001) on the response. Factor D (microwave power) also shows a significant influence (*p* < 0.05). Based on the magnitude of their F-values, the relative impact of the factors on tea saponin extraction yield follows this order: liquid-to-solid ratio (A) > ethanol concentration (B) > extraction time (C) > microwave power (D) [55]. The liquid-to-solid ratio exhibited the most significant impact, which may be attributed to the higher saponin content in the JJ samples compared to those from GZ. Under these conditions, the driving force of the mass transfer process becomes the primary limiting factor. Ethanol concentration was the second most influential parameter, as it determines solvent polarity. Maximum extraction yield is achieved only when the polarity matches that of saponins such as tea saponin; any deviation from the optimal concentration results in reduced efficiency. Microwave power exerted less influence than extraction time, likely because the lower structural polymer content (lignin, cellulose, and hemicellulose) in JJ husks rendered cell walls more susceptible to microwave disruption. Consequently, even the lowest microwave setting applied was sufficient to effectively disintegrate the cellular structure of *C. oleifera* husks [58].

**Figure 11:**
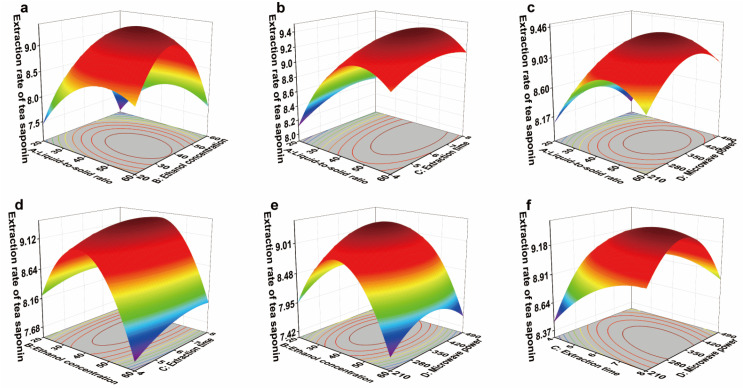
Response surface graph of the effects of liquid-to-solid ratio (A), ethanol concentration (B), extraction time (C), and microwave power (D) on tea saponin extraction yield from *C. oleifera* husks in the JJ region. (**a**) AB; (**b**) AC; (**c**) AD; (**d**) BC; (**e**) BD; (**f**) CD. The peaks of the surface are usually colored yellow/red to indicate a high response value. The low-lying areas of the curved surface are usually colored blue/green to indicate a low response value.

The response surface plot for the tea saponin extraction yield from JJ samples is shown in Figure 11. A steeper slope (higher inclination) on the 3D surface indicates a more significant interaction between factors. Concurrently, an intensifying surface color corresponds to a sharper increase in the extraction yield [61].

Based on the data in Table 8, the interaction between liquid-to-solid ratio and ethanol concentration has a significant effect on tea saponin extraction yield. The interaction between ethanol concentration and the liquid-to-solid ratio reflects their combined influence on both the solubility of tea saponin in ethanol and the mass transfer efficiency between the solid and liquid phases. When a strong mass transfer driving force is coupled with low solubility, or when high solubility is constrained by limited mass transfer, the two factors exhibit an antagonistic effect. Conversely, when solubility and mass transfer are optimally balanced, the solvent can penetrate the cells more effectively, allowing tea saponin to be rapidly and thoroughly released, thereby producing a synergistic effect and maximizing extraction efficiency [63]. The interactions between the liquid-to-solid ratio and microwave power, as well as between extraction time and microwave power, exert highly significant effects on tea saponin yield. The liquid-to-solid ratio dictates the physical characteristics of the reaction system and its energy absorption efficiency, while microwave power provides the energy input. Together, they determine the effective energy density per unit volume and the overall mass transfer efficiency. Changes in microwave power can markedly modify the impact of the liquid-to-solid ratio. Moreover, microwave power controls the rate of energy delivery, whereas extraction time defines the duration of energy application; collectively, they regulate the total energy input and the balance of thermal and non-thermal effects. To some extent, comparable extraction outcomes can be achieved either by lowering power and extending time or by increasing power and shortening the extraction period.

Optimization of the extraction yield of *C. oleifera* husks in the JJ region using response surface methodology (RSM) yielded the following theoretical optimum conditions: liquid-to-solid ratio of 47.44, ethanol concentration of 37.28%, extraction time of 6.06 min, and microwave power of 360.49 W. Under these conditions, the predicted extraction yield was 9.40%.

To validate the theoretical optimum while accounting for practical constraints, the process parameters were adjusted as follows: liquid-to-solid ratio of 47.44, ethanol concentration of 37.28%, extraction time of 6 min, and microwave power of 350 W. Validation experiments conducted with these modified parameters resulted in an actual tea saponin extraction yield of 9.39 ± 0.02%. This represents a relative error of merely 0.18% compared to the theoretical prediction, confirming the model’s validity in representing the actual extraction process [60].

## 4. Conclusions

This study systematically characterized the physicochemical and structural properties of *Camellia oleifera* husks from three representative regions of Jiangxi Province and optimized tea saponin extraction yield using microwave-assisted solvent extraction. Significant regional variations were observed, with YC husks exhibiting higher saponin content (16.29 ± 0.02%), lower cellulose (21.05 ± 1.05%) and lignin (12.48 ± 1.14%) levels, and higher hemicellulose (27.40 ± 0.80%), while JJ husks displayed greater porosity and specific surface area (40–60 mesh, 4.15 ± 0.04 m^2^/g). Single-factor experiments and response surface methodology revealed that ethanol concentration and liquid-to-solid ratio were the most influential factors, followed by microwave power and extraction time. The optimized extraction conditions for GZ, YC, and JJ yielded tea saponin extraction yields of 7.49 ± 0.01%, 16.29 ± 0.02%, and 9.39 ± 0.02%, respectively, with minimal deviation from predicted values, confirming model reliability. These findings provide novel insights into the structure–function relationship of *C. oleifera* husks and establish a practical basis for efficient, sustainable extraction of bioactive compounds. The results highlight the potential of agricultural by-product valorization to promote resource recycling, economic benefits, and green development in the *Camellia oleifera* industry. Moreover, MASE faces limitations for several reasons. The technology requires expensive equipment capable of withstanding high pressure, leading to high energy consumption and maintenance costs. Its rapid heating can cause local overheating, degrading heat-sensitive components like saponin. Furthermore, it is only effective with polar solvents (e.g., ethanol, water), restricting its use with non-polar solvents (e.g., n-hexane). Safety concerns also arise from the use of flammable solvents under pressure, coupled with the need for shielding against electromagnetic radiation.

## Figures and Tables

**Table 1:** Factors and levels used in the Box–Behnken response surface optimization experiment.

Experimental Factors	Label	Level		
		−1	0	1
Liquid-to-solid ratio (mL/g)	A	20	40	60
Ethanol concentration (%)	B	20	40	60
Extraction time (min)	C	4	6	8
Microwave power (W)	D	210	350	490

**Table 2:** Elemental analysis of *C. oleifera* husks.

The origin of *C. oleifera* husks	Elemental Analysis (%)				
	C	H	O	N	S
GZ	46.24 ± 0.07 ^b^	5.72 ± 0.03 ^a^	47.61 ± 0.11 ^b^	0.42 ± 0.02 ^a^	0.02 ± 0.00 ^b^
YC	45.76 ± 0.03 ^c^	5.68 ± 0.01 ^b^	48.14 ± 0.03 ^a^	0.41 ± 0.01 ^ab^	0.02 ± 0.00 ^b^
JJ	46.97 ± 0.06 ^a^	5.71 ± 0.01 ^a^	46.88 ± 0.08 ^c^	0.39 ± 0.01 ^b^	0.06 ± 0.01 ^a^

Values in the same column with different superscripts (a–c) are significantly different (*p* < 0.05).

**Table 3:** Pore size parameters of *C. oleifera* husk samples of 40–60, 60–80, and 80–100 mesh in three regions.

Sample (Mesh Size)	BET Surface Area (m^2^/g)	Pore Volume (cm^3^/g)	Average Pore Size (nm)	Most Frequent Pore Diameter (nm)
GZ40~60	1.57 ± 0.02 ^c^	0.004 ± 0.00	9.66 ± 0.04 ^f^	1.88 ± 0.01 ^a^
GZ60~80	1.28 ± 0.01 ^e^	0.003 ± 0.00	10.22 ± 0.05 ^e^	1.83 ± 0.01 ^b^
GZ80~100	1.23 ± 0.01 ^f^	0.004 ± 0.00	13.46 ± 0.07 ^a^	1.58 ± 0.01 ^c^
YC40~60	1.53 ± 0.02 ^d^	0.004 ± 0.00	10.75 ± 0.06 ^d^	1.86 ± 0.01 ^ab^
YC60~80	1.48 ± 0.01 ^d^	0.005 ± 0.00	12.63 ± 0.06 ^c^	1.85 ± 0.01 ^ab^
YC80~100	1.50 ± 0.01 ^d^	0.005 ± 0.00	12.67 ± 0.06 ^c^	1.57 ± 0.01 ^c^
JJ40~60	4.15 ± 0.04 ^a^	0.007 ± 0.00	6.77 ± 0.04 ^h^	1.86 ± 0.01 ^ab^
JJ60~80	3.41 ± 0.03 ^b^	0.006 ± 0.00	7.31 ± 0.05 ^g^	1.86 ± 0.01 ^ab^
JJ80~100	0.89 ± 0.01 ^g^	0.003 ± 0.00	12.95 ± 0.07 ^b^	1.59 ± 0.01 ^c^

Values in the same column with different superscripts (a–h) are significantly different (*p* < 0.05).

**Table 4:** Box–Behnken experimental design and results for the extraction yield of tea saponins in GZ.

Test Number	A	B	C	D	Extraction Yield of Tea Saponins (%)
1	20	20	6	350	6.36
2	40	40	4	210	7.10
3	60	40	6	210	5.31
4	40	60	6	210	6.11
5	40	40	6	350	6.13
6	40	20	8	350	6.25
7	20	40	4	350	6.42
8	40	40	6	350	6.47
9	60	40	8	350	6.30
10	60	60	6	350	6.95
11	20	40	8	350	6.48
12	40	40	6	350	7.28
13	60	40	4	350	6.21
14	40	20	6	210	5.22
15	20	40	6	210	6.31
16	40	40	8	490	5.27
17	40	20	4	350	6.27
18	20	60	6	350	6.98
19	60	20	6	350	6.44
20	60	40	6	490	7.03
21	40	40	4	490	6.17
22	40	40	8	210	5.18
23	40	60	4	350	6.38
24	40	60	8	350	5.37
25	40	20	6	490	7.44
26	20	40	6	490	7.21
27	40	60	6	490	7.26

**Table 5:** Analysis of variance of the Box–Behnken experimental design in GZ.

Source	Sum of Squares	df	Mean Square	F-Value	*p*-Value Prob > F
Model	11.43	14	0.82	211.04	<0.0001 ^b^
A—Liquid-to-solid ratio	1.53	1	1.53	396.57	<0.0001 ^b^
B—Ethanol concentration	3.07	1	3.07	793.93	<0.0001 ^b^
C—Extraction time	0.024	1	0.024	6.28	0.0276 ^a^
D—Microwave power	0.17	1	0.17	43.45	<0.0001 ^b^
AB	9.00 × 10^−4^	1	9.00 × 10^−4^	0.23	0.6382
AC	3.60 × 10^−3^	1	3.60 × 10^−3^	0.93	0.3537
AD	5.63 × 10^−3^	1	5.63 × 10^−3^	1.45	0.2511
BC	6.25 × 10^−4^	1	6.25 × 10^−4^	0.16	0.6947
BD	1.00 × 10^−4^	1	1.00 × 10^−4^	0.026	0.8749
CD	1.23 × 10^−3^	1	1.23 × 10^−3^	0.32	0.5839
A^2^	0.03	1	0.03	7.76	0.0165 ^a^
B^2^	5.63	1	5.63	1455.95	<0.0001 ^b^
C^2^	1.48	1	1.48	381.92	<0.0001 ^b^
D^2^	1.22	1	1.22	316.08	<0.0001 ^b^
Residual	0.046	12	3.87 × 10^−3^		
Lack of fit	0.017	10	1.71 × 10^−3^	0.12	0.9931
Pure error	0.029	2	0.015		
Cor total	11.47	26			

Note: ^a^: *p* < 0.05 was considered statistically significant, ^b^: *p* < 0.0001 was regarded as extremely significant.

**Table 6:** Box–Behnken experimental design and results of extraction yield of tea saponins in YC.

Test Number	A	B	C	D	Extraction Yield of Tea Saponin (%)
1	20	20	6	350	15.03
2	40	40	4	210	14.75
3	60	40	6	210	13.78
4	40	60	6	210	15.24
5	40	40	6	350	14.41
6	40	20	8	350	14.73
7	20	40	4	350	14.75
8	40	40	6	350	15.27
9	60	40	8	350	14.98
10	60	60	6	350	15.34
11	20	40	8	350	15.02
12	40	40	6	350	16.18
13	60	40	4	350	14.31
14	40	20	6	210	13.91
15	20	40	6	210	14.88
16	40	40	8	490	14.22
17	40	20	4	350	14.66
18	20	60	6	350	15.32
19	60	20	6	350	14.98
20	60	40	6	490	15.74
21	40	40	4	490	14.18
22	40	40	8	210	14.13
23	40	60	4	350	14.92
24	40	60	8	350	14.27
25	40	20	6	490	16.15
26	20	40	6	490	15.96
27	40	60	6	490	16.29

**Table 7:** Analysis of variance of the Box–Behnken experimental design in YC.

Source	Sum of Squares	df	Mean Square	F-Value	*p*-Value Prob > F
Model	12.37	14	0.88	74.09	<0.0001 ^c^
A—Liquid-to-solid ratio	1.41	1	1.41	118.65	<0.0001 ^c^
B—Ethanol concentration	0.53	1	0.53	44.39	<0.0001 ^c^
C—Extraction time	0.5	1	0.5	42.3	<0.0001 ^c^
D—Microwave power	0.58	1	0.58	48.72	<0.0001 ^c^
AB	0.76	1	0.76	63.49	<0.0001 ^c^
AC	2.50 × 10^−3^	1	2.50 × 10^−3^	0.21	0.6552
AD	0.16	1	0.16	13.42	0.0032 ^b^
BC	0.017	1	0.017	1.42	0.2568
BD	0.09	1	0.09	7.55	0.0177 ^a^
CD	1.00 × 10^−2^	1	1.00 × 10^−2^	0.84	0.3778
A^2^	0.28	1	0.28	23.84	0.0004 ^b^
B^2^	7.13	1	7.13	597.63	<0.0001 ^c^
C^2^	2.69	1	2.69	226.04	<0.0001 ^c^
D^2^	1.83	1	1.83	153.53	<0.0001 ^c^
Residual	0.14	12	0.012		
Lack of fit	0.088	10	8.82 × 10^−3^	0.32	0.9109
Pure error	0.055	2	0.027		
Cor total	12.51	26			

Note: ^a^: *p* < 0.05 was considered statistically significant; ^b^: *p* < 0.01 was regarded as highly significant; ^c^: *p* < 0.0001 was regarded as extremely significant.

**Table 8:** Box–Behnken experimental design and results of extraction yield of tea saponins in JJ.

Test Number	A	B	C	D	Extraction Yield of Tea Saponins (%)
1	20	20	6	350	7.46
2	40	40	4	210	8.28
3	60	40	6	210	7.18
4	40	60	6	210	7.72
5	40	40	6	350	8.42
6	40	20	8	350	9.01
7	20	40	4	350	8.84
8	40	40	6	350	8.86
9	60	40	8	350	8.07
10	60	60	6	350	8.66
11	20	40	8	350	7.91
12	40	40	6	350	8.92
13	60	40	4	350	8.23
14	40	20	6	210	7.63
15	20	40	6	210	8.37
16	40	40	8	490	8.02
17	40	20	4	350	8.15
18	20	60	6	350	8.88
19	60	20	6	350	8.43
20	60	40	6	490	9.01
21	40	40	4	490	7.90
22	40	40	8	210	7.48
23	40	60	4	350	8.08
24	40	60	8	350	7.61
25	40	20	6	490	9.35
26	20	40	6	490	9.24
27	40	60	6	490	9.37

**Table 9:** Analysis of variance of the Box–Behnken experimental design in JJ.

Source	Sum of Squares	df	Mean Square	F-Value	*p*-Value Prob > F
Model	9.97	14	0.71	214.02	<0.0001 ^c^
A—Liquid-to-solid ratio	1.52	1	1.52	456.68	<0.0001 ^c^
B—Ethanol concentration	0.6	1	0.6	179.9	<0.0001 ^c^
C—Extraction time	0.2	1	0.2	60.18	<0.0001 ^c^
D—Microwave power	0.039	1	0.039	11.58	0.0052 ^b^
AB	0.02	1	0.02	5.89	0.0319 ^a^
AC	5.63 × 10^−3^	1	5.63 × 10^−3^	1.69	0.2179
AD	0.044	1	0.044	13.25	0.0034 ^b^
BC	0.016	1	0.016	4.70	0.0511
BD	6.25 × 10^−4^	1	6.25 × 10^−4^	0.19	0.6724
CD	0.08	1	0.08	24.41	0.0003 ^b^
A^2^	1.55	1	1.55	466.72	<0.0001 ^c^
B^2^	6.79	1	6.79	2040.84	<0.0001 ^c^
C^2^	0.11	1	0.11	32.36	0.0001 ^c^
D^2^	0.87	1	0.87	260.77	<0.0001 ^c^
Residual	0.04	12	3.33 × 10^−3^		
Lack of fit	0.03	10	3.01 × 10^−3^	0.61	0.7554
Pure error	9.80 × 10^−3^	2	4.90 × 10^−3^		
Cor total	10.01	26			

Note: ^a^: *p* < 0.05 was considered statistically significant; ^b^: *p* < 0.01 was regarded as highly significant; ^c^: *p* < 0.0001 was regarded as extremely significant.

## Data Availability

The data presented in this study are available upon request from the corresponding author. The data are not publicly available due to privacy restrictions.
